# A Parametric Time-to-Event Modelling of Recurrent Ischemic Stroke After Index Stroke Among Patients With and Without Diabetes Mellitus: Implementation of Temporal Validation of the Model

**DOI:** 10.7759/cureus.50794

**Published:** 2023-12-19

**Authors:** Marwa Elhefnawy, Sabariah Noor Harun, Teo Leykhim, Balamurgan Tangiisuran, Hadzliana Zainal, Irene Looi, Norsima Sidek, Zariah Abdul Aziz, Siti Maisharah Sheikh Ghadzi

**Affiliations:** 1 Clinical Pharmacy, School of Pharmaceutical Sciences, Universiti Sains Malaysia, Penang, MYS; 2 Clinical Research Centre, Hospital Pulau Pinang, Penang, MYS; 3 Neurology Unit, Hospital Seberang Jaya, Penang, MYS; 4 Clinical Research Centre, Hospital Sultanah Nur Zahirah, Terengganu, MYS

**Keywords:** ischemic stroke, nonmem, diabetes, recurrent ischemic stroke, time to event

## Abstract

Objectives

Compared with the first stroke, neurological impairment caused by stroke recurrence is more serious, more difficult to treat, and has a higher mortality rate, especially among ischemic stroke (IS) patients with diabetes mellitus (DM). Although there are established correlations between factors and IS recurrence, there were some issues regarding the naive hazard of IS recurrence with no risk factor influence, and how does the baseline hazard differ among patients with DM and non-DM? To answer all these questions, two time-to-event (TTE) models of recurrent IS after the index IS were developed among IS patients with DM and non-DM.

Method

A total of 7697 patients with an index IS attack were extracted from the Malaysian Registry of Neurology and stratified according to DM status. Several parametric survival models were evaluated using nonlinear mixed-effect modeling software (NONMEM 7.5). The final model was determined according to the lowest objective function value, graphical evaluation, numerical diagnostics, and clinical plausibility. Additionally, the final model was validated internally and temporally using Kaplan-Meier visual predictive checks (KM-VPCs).

Results

One hundred ninety-five (5.82%) of 3493 DM patients and 138 (3.28%) of 4204 non-DM patients developed a recurrent IS with a maximum follow-up of 7.37 years. Gompertz’s model best fitted the data. With no influence on risk factors, the index IS attack was predicted to contribute to the hazard of recurrent IS by 0.356 and 0.253 within the first six months after the index IS among patients with and without DM, respectively. Even after six months of index IS, the recurrent IS baseline hazard was not equal to zero among both groups (0.0023, 0.0018). Moreover, after incorporating the time and risk factors, the recurrent hazards increased exponentially during the first three years after the index IS followed by an exponential reduction afterward. The recurrent IS predictors among DM patients were ischemic heart disease (IHD) and hyperlipidemia (HPLD). IHD and HPLD increased the hazard of recurrent IS by 2.40 and 1.88 times, respectively, compared to those without IHD and HPLD before index IS (HR, 2.40 (1.79-3.20)), and (HR, 1.88 (1.44-2.45)) respectively.

Conclusion

The baseline hazard was the highest during the first six months after the index IS. Moreover, receiving medications for secondary prevention failed to demonstrate a significant association with reducing IS recurrence among IS patients with DM, suggesting a need for more intensive patient screening and new strategies for secondary prevention among IS patients with DM.

## Introduction

Stroke is one of the major causes of handicap and mortality worldwide [1]. Patients surviving an initial ischemic stroke (IS) are known to be at significantly increased risk for recurrent strokes [2-5]. A meta-analysis of 58 studies (n=131.299) with a mean follow-up of 3.5 years (range 1.0-10.0) showed that the risk of recurrent stroke after the first IS was 4.26 percent/year [5].

Diabetes is a pandemic chronic disease causing widespread disability and death worldwide [6,7]. In 2017, the prevalence of type 2 diabetes mellitus was 6,059/100,000, with approximately 462 million people affected worldwide [8]. Additionally, by 2030, diabetes is predicted to reach 4.4% in all age groups [6,7]. Stroke patients with a prior history of diabetes are known to have a bad prognosis and a higher risk of recurrent stroke with a high mortality rate compared to those without diabetes mellitus (DM) [9,10]. A previous meta-analysis of 14 studies reported an increased hazard ratio (HR) of recurrent IS (HR: 1.44 (95% CI: 1.28-1.61)) among patients with DM [11]. A more recent study found that within 3.25 years after IS, patients with DM have a higher risk of recurrent vascular events than patients without diabetes (the adjusted hazard ratio of diabetes was 1.50 (95% CI: 1.08-2.10)). [12]. Additionally, among the 14,526 ischemic stroke patients in the China National Stroke Registry, there were higher frequencies of recurrent stroke at the three- and six-month time points among diabetics compared to non-diabetics [13]. Recurrent neurological damage is known to be harder to control and has a higher mortality rate compared to the first stroke [14]. The data on secondary prevention strategies among IS patients with DM is still inconclusive. These strategies may be insufficient, and additional care with improved secondary preventative treatment for patients with DM should be considered [15]. The influence of each risk factor on the baseline hazard of IS recurrence among patients with DM needs to be investigated and quantified to tailor better preventive strategies. What if the risk factors for recurrent IS are removed, will the hazard among patients with DM still be higher than non-DM? Moreover, the comparison of the hazard trend of recurrence between patients with DM and those without DM needs to be investigated.

Information on the recurrent IS distribution at different time points after the index IS is limited. Moreover, studies using the parametric approach are still lacking on this topic [16]. The previous studies on the prognosis of recurrent IS among DM patients used Cox-regression analysis, which is a semi-parametric survival method of analysis. The Cox model includes the covariate effect on the hazard without quantifying the baseline hazard of recurrent stroke or the distribution of the hazard function. In the case of recurrent IS, the baseline hazard is defined as the hazard of having recurrent IS among patients with DM after the index IS without the influence of any risk factors, while the distribution hazard function is used to quantify the likelihood of IS recurrence at different time points. Unlike Cox-regression, the parametric approach to survival analysis can quantify the baseline hazard and distribution hazard function of the recurrent IS. This enables more time-dependent prognostic information that positively influences disease follow-up strategies.

Thus, this study aimed to quantify the baseline hazard as well as the hazard pattern after adding significant risk factors for recurrent ischemic stroke through the development of two time-to-event (TTE) models of recurrent IS among patients with and without DM using a parametric approach.

## Materials and methods

Data collection

The data from the patients with IS history from August 2009 to December 2016 was extracted from the Malaysian Registry of National Neurology (NNEUR), which includes all hospitals in 13 states in Malaysia. This data was further stratified according to DM status. This study is considered a secondary analysis of the data from the Malaysian NNEUR. The information on the Malaysian National Stroke Registry was included in previously published papers [9,10,17]. DM was identified in the registry database either by physician diagnosis, antidiabetic medication history, patients’ electronic records, or the antidiabetic medications prescribed during discharge. The diagnosis of stroke was made based on the World Health Organization’s criteria [18]. The diagnosis was verified through magnetic resonance imaging or brain computed tomography. Patients with index IS were the patients who registered with the first IS into the NNEUR from 2009 to 2016. While those with recurrent IS were identified as patients with any IS recorded in the NNEUR database after the index IS. The NNEUR has been described comprehensively in previously published research [19]. Patients' demographic data and concurrent disease data, including HPLD, hypertension (HTN), IHD, hyperuricemia, atrial fibrillation (AF), and medications for secondary prevention or concurrent disease control, were investigated.

Ethics approval

This study was approved ethically by the Committee of Medical Research and Ethics, Ministry of Health, Malaysia, under Research ID: NMRR-08-1631-3189. Informed consent has been obtained from participants.

Data for the temporal validation

Temporal validation is the same idea as external validation. Both of them estimate the final developed model's predictive performance. The data used for the temporal validation was extracted from patients in similar settings to those in which the original model was developed. At the same time, external validation refers to the process of checking the performance of the final h=h0∗exp(βt+βcov(cov)) developed model on data from centers different from those included in the original model development [20]. The temporal validation of the final model was performed on the data from another cohort of patients with DM and index IS history extracted from January 2017 until December 2020 in the NNEUR. 

Model development

Time-to-recurrent IS events and their predictors among IS patients with and without DM were estimated using NONMEM version 7.5 software through the LAPACE (ADVAN=6 TOL=9 NSIG=3) estimation method, and Perl speaks NONMEM (PsN) version 4.1.0. The event was described as getting recurrent IS events after the index IS. All event times had been dealt with as exact time models; thus, the event was supposed to occur at the time of observation. The last observation time (end of follow-up) was recorded and treated as a censored observation. The two TTE model developments (among patients with and without DM) involved two steps: (i) base model development and (ii) exploration of potential covariates.

Base Model Development

According to Equation (1), a parametric survival function was used to describe the recurrent IS between patients with and without DM in the two TTE models.

S(t)=$exp-\int_{0}^{t}h(t)dt$ (Equation 1)

Where h(t) is the hazard and S(t) is the survival, which is defined as the probability of not having any recurrent IS within a specific time. S(t) is described as a function of the cumulative hazard within a period from zero to time t.

For base model selection, different functions for the hazard h(t) were explored, starting from a simple time-independent constant hazard to more complicated functions, namely, Gompertz and Weibull [21]. These three functions were investigated according to the following equations (2), (3), and (4), respectively:

 $h=h_{0} &times;exp 0$ (Equation 2)

 $h=h_{0} &times;exp \beta t$ (Equation 3)

 $h=h_{0} &times;exp \beta ln (t)$ (Equation 4) 

The baseline hazard of recurrent IS during two different time intervals was quantified, and the hazard description was added at different time intervals. The baseline hazard h0(t) was quantified by inserting different time intervals, where h0 equals θ1 if time < 0.5 years and θ3 if time ≥ 0.5 years. β, which represents the shape of the time distribution or coefficient of factor time, considered as θ2 when time < 3 years, and θ4 when time ≥ 3 years, indicates the hazard changes at a different time interval after the index IS.

Covariate Model Development and Model Evaluation

The potential risk factors that may influence the hazard of recurrent IS among IS patients with and without DM were explored with the best-developed base model by incorporating each covariate in the hazard function. A parameter, βcov, for each covariate was estimated using Equation 5.

 $h=h_{0} *exp (\beta t+\beta cov(cov)$) (Equation 5)

Where h0 is the baseline hazard, and βt is the shape parameter of the Gompertz hazard function. The hazard ratio (HR) is calculated by exponentiating the explanatory variable coefficient. The HR of the explanatory variable reflects its influence on the hazard related to the hazard when this explanatory variable is unpresented. Initially, the covariates were tested univariately, i.e., each covariate relationship was identified on the base hazard. Then, based on the results, covariate relationships were evaluated for a systematic covariate search through a stepwise analysis approach, i.e., forward stepwise inclusion followed by backward elimination [22]. The covariate is considered significant when the objective function value (OFV) decreases by at least 3.84 (p<0.05) for one degree of freedom (df) (addition of one covariate parameter) in the forward selection. While in the backward deletion, the significant value was set to p< 0.01, representing an increase in the OFV of at least 6.64 to be retained in the model for one df [22].

The predictive performance of the final model was evaluated to ensure that the model describes the data sufficiently. This was done through Kaplan-Meier visual predictive checks (VPCs) for internal and temporal validation. For the VPC simulation, additional time points were added to the time points with no clinical observations in the original dataset until 7.37 years. The covariate model development and model evaluation were described comprehensively in a previously published preprint [23].

Temporal validation of the final TTE model among IS patients with DM

Data from 1262 DM patients with and without recurrent IS were used to perform temporal validation for the final developed model. For the VPC plotting, the final model was utilized to simulate 1000 replicates of the dataset. By using the new set of data for validation, the model's ability to predict the probability of not having recurrent IS among DM patients was tested to evaluate the predictive performance of the developed model by overlying the plot of VPC on the validation data Kaplan-Meier curve.

## Results

From August 2009 to December 2016, out of 3493 patients with DM, 195 patients (5.55%) had a recurrent IS within 7.37 years of follow-up. While among 4204 patients without DM, 138 patients (3.28%) developed recurrent IS (Table 1). This study included all categories of age, from young to older, with a median of 62.9 years at the index IS time. As shown in Table 1, most of the DM patients who developed recurrent IS were males (99, 51.03%), while most of the non-DM patients were females (38.40%). The greater amount of data in the database was from East Malaysian hospitals; hence, a higher number of patients belonged to other ethnicities. The percentage of smokers who developed recurrent IS among patients with and without DM was 57.94% and 64.49%, respectively. While 180 DM patients (92.30%) and 108 patients (78.26%) with non-DM were hypertensive before index IS, the number of IHD cases among DM patients who developed recurrent IS was 52 (26.66%), while this number decreased among patients with non-DM to be 25 (18.11%). The number of patients who had HPLD before index IS was 96 (49.23%) and 63 (45.65%) among patients with and without DM, respectively.

**Table 1:** Characteristics of ischemic stroke (IS) patients with (N = 3493) and without (N = 4204) DM after follow-up of a maximum of 7.37 years after index IS ACEI: angiotensin-converting enzyme inhibitors; ADM: antidiabetics; AF: atrial fibrillation; APLT: antiplatelet; BB: beta-blockers; CCB: calcium channel blockers; DIU: diuretics; DM: diabetes mellitus, FHOS: family history of stroke; IHD: ischemic heart disease; HTN: hypertension; HPLD: hyperlipidemia; HU: hyperuricemia; NIHSS: National Institute of Health Stroke Scale; N: number of patients

Variable	Diabetic patients with recurrent IS N=195 (%)	Diabetic patients with no recurrent IS N=3298 (%)	Non-diabetic patients with recurrent IS N=138 (%)	Non-diabetic patients with no recurrent IS N=4066 (%)
Age group				
≤60	92(47.17)	1317(39.93)	58(42.02)	1607(39.54)
>60	103(52.82)	1981(60.06)	80(57.97)	2459(60.45)
Female	101(51.79)	1647(49.93)	53(38.40)	1607(39.52)
Ethnicity				
Malay	80(41.02)	697(21.13)	75(54.34)	782(19.23)
Chinese	6(3.07)	87(2.63)	2(1.44)	118(2.90)
Indian	3(1.53)	50(1.51)	-	30(0.737)
Others	106(54.35)	2464(74.71)	61(44.20)	3137(77.15)
Smoker	113(57.94)	1630(49.42)	89(64.49)	1917(47.14)
Duration of diabetes (years)			-	-
<1	9(4.61)	340(10.30)		-
1-5	97(40.51)	1676(50.81)		-
6-10	42(21.53)	521(15.79)		-
>10	43(22.05)	765(23.19)		-
Family history of stroke	16(8.20)	152(4.60)	11(7.97)	247(6.07)
HTN	180(92.30)	2863(86.81)	108(78.26)	2355(57.91)
HTN Duration (years)				
≤5	94(48.20)	1660(50.33)	69(50)	1507(37.06)
>5	86(44.10)	1203(36.47)	39(28.26)	848(20.85)
IHD	52(26.66)	420(12.73)	25(18.11)	382(9.39)
HPLD	96(49.23)	1004(30.44)	63(45.65)	865(8.97)
Atrial fibrillation	4(2.05)	87(2.63)	5(3.62)	172(4.23)
Hyperuricemia	10(5.12)	121(3.66)	6(4.34)	97(2.38)
NIHSS				
Minor	81(41.53)	1535(46.54)	64(46.37)	1873(46.06)
Moderate/severe	114(58.46)	1763(53.45)	74(53.62)	2193(53.93)
Received medication for concurrent disease control and/or secondary prevention				
Antihyperlipidemic	167(85.64)	2926(88.72)	120(86.95)	3682(90.55)
APLT	167(85.64)	2978(90.29)	118(85.50)	3635(89.39)
ACEI	61(31.28)	1144(34.68)	37(26.81)	1154(28.38)
BB	24(12.30)	407(12.34)	15(10.86	343(8.43)
ADM	117(60)	2005(60.79)	15(10.86	301(7.40)
DIU	22(11.28)	255(7.73)	7(5.07)	170(4.18)
CCB	59(30.25)	784(23.77)	21(15.21)	736(18.10)

The percentage of recurrent IS patients who received antihyperlipidemics for secondary prevention among patients with and without DM was 85.64% and 86.95%, respectively. The number of patients who received antiplatelet (APLT) for secondary prevention was 167 (85.64%) among patients with DM and 118 (85.50%) among patients with non-DM.

Final developed TTE model among ischemic stroke patients with DM and non-DM

Baseline Hazard During Different Time Intervals Among Patients With DM and non-DM

**Figure 1:**
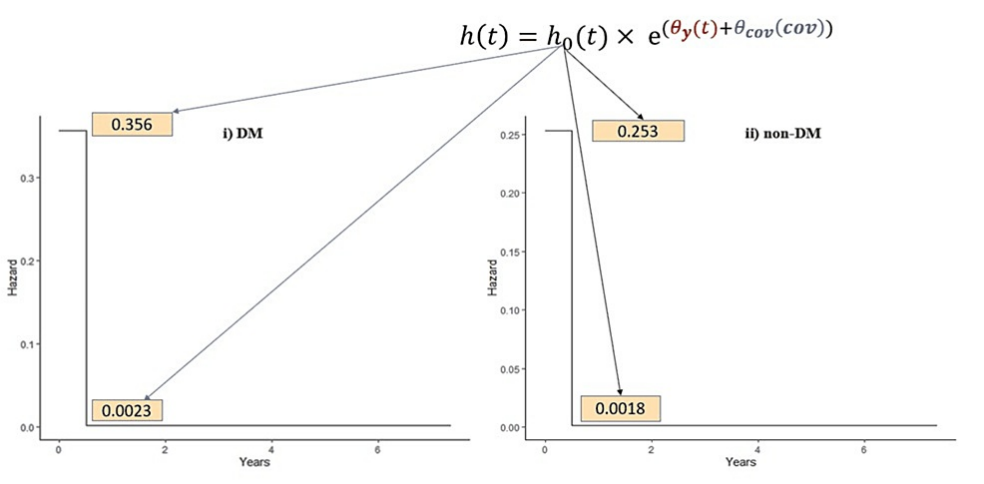
Baseline hazard among patients with i) DM and ii) non-DM during and after the first six months of ischemic stroke

Among the investigated TTE models, the Gompertz model showed the best fit to our data according to OFV (Table 2). As shown in Figure 1, the recurrent IS hazard among DM and non-DM patients with no influence of any significant predictors within the first six months after the index IS was 0.356 and 0.253, respectively. Even after six months of index IS, the baseline hazard of recurrent IS was not equal to zero among both groups (0.0023, 0.0018).

**Table 2:** Objective function value and the number of parameters in different tested survival models ∆OFV: objective function value difference; h: hazard; h0: baseline hazard; t: time. After inserting time intervals, h0 equals θ1 if time < 0.5 years, θ3 if time ≥ 0.5; θy equals θ2 if time < 3 years, θ4 if time ≥ 3 years. Significance: p-value < 0.05. Highlighted in blue/yellow are selected base models with the lowest OFV

Number of parameters	Variable	Model	OFV among patients with DM	∆OFV among patients with DM	p-Value	OFV among patients with non-DM	∆OFV among patients with non-DM	p-Value
1	Constant	h(t)=h_0_	2103.088	0		1681.446	0	
2	Gompertz	h(t)=h_0_ × e^(θ_2_)t^	2084.082	-19.006	<0.0001	1672.202	-9.244	0.0023
2	Weibull	h(t)=h_0_ × e^(θ_2_)ln(t)^	2091.736	-11.35	0.0007	1677.389	-4.057	0.043
After inserting different time intervals								
4	Gompertz	h(t)=h_0_ × e^(θ_y_)t^	1522.99	-580.09	<0.0001	1259.045	-442.401	<0.0001
4	Weibull	h(t)=h_0_ × e^(θ_y_)ln(t)^	1893.6103	-209.47	<0.0001	1503.139	-178.307	<0.0001

Hazard Pattern During Different Time Intervals (Final TTE Model 1&2)

After incorporating the factor of time and the established risk factor, the recurrent hazard increased exponentially during the first three years after the index IS and then exponentially reduced afterwards (Table 3).

**Table 3:** Estimated parameters of the final model for recurrent IS after index IS among patients with and without DM h: baseline hazard; RSE: relative standard error; 95% CI: 95% confidence interval; ⍺: shape parameter; aHR: adjusted hazard ratio; HTN: hypertension; HPLD: hyperlipidemia; IHD: ischemic heart disease. The RSE (%) was obtained from the sampling importance resampling (SIR) method.

Parameter		Description	Typical value of final TTE among patients with DM	Half-life (Ln2/⍺)	aHR 95%CI	RSE%		Typical value of final TTE among patients with non-DM	Half-life (Ln2/⍺)	aHR 95%CI	RSE%
θ_1_ (<6months)	θ_1_	Baseline hazard	0.356	-	-	13.69%	θ_1_	0.253	-	-	24.38%
θ_3_( ≥6months)	θ_3_	Baseline hazard	0.0023	-	-	17.01%	θ_3_	0.0018	-	-	23.09%
⍺ (<3)	θ_2_	Shape parameter in the first three years after index IS	1.58	0.43 (5.25 months)	-	5.98%	θ_2_	1.7	0.40 (4.88 months)	-	6.37%
⍺ (≥3)	θ_4_	Shape parameter after three years of index IS	0.242	2.85 years	-	22.36%	θ_4_	0.213	3.24years	-	33.07%
IHD (covariate)	θ_5_	Effect of baseline IHD on hazard	0.876	-	2.40(1.79-3.20)	16.88%	-	-	­-	­-	-
HPLD (covariate)	θ_6_	Effect of baseline HPLD on hazard	0.633	-	1.88(1.44-2.45)	21.38%	θ_5_	1.03		2.801(2.00-3.90)	16.51%
HTN (covariate)	-	Effect of baseline HTN on hazard	-	-	-	-	θ_6_	0.789		2.201(1.53-3.14)	23.17%
Antihyperlipidemic	-	Effect of baseline antihyperlipidemic on hazard	-	-	-	-	θ_7_	-0.835		0.433(0.65-0.28)	24.80%

Covariates Influencing the Hazard of Recurrent IS After Index IS Among Patients With DM and Non-DM (Final TTE Model 1&2)

**Figure 2:**
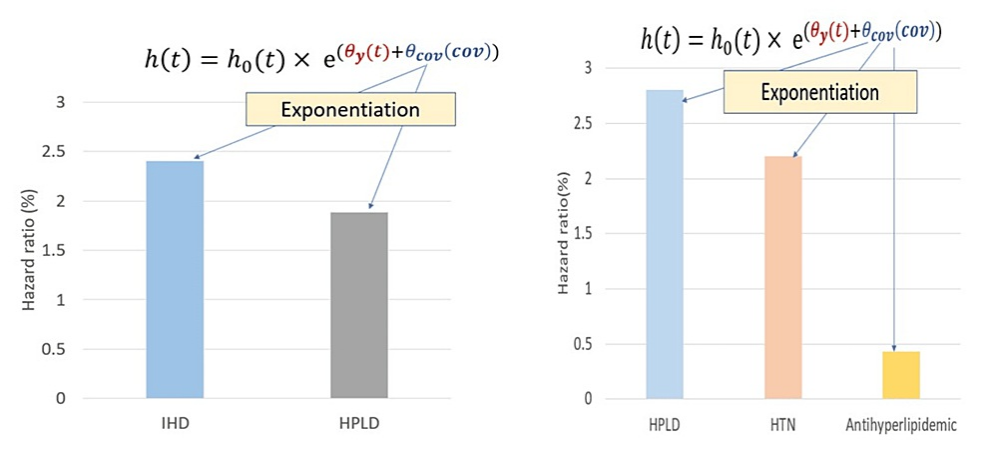
Significant covariates influence the hazard of recurrent IS among patients with (left panel) and without (right panel) DM. IHD: ischemic heart disease; HPLD: hyperlipidemia; HTN: hypertension

The results of the final TTE model among patients with DM are shown in Table 3, and it can be observed that the recurrent IS rate among DM patients with IHD was 2.40 times higher than that in patients with no-IHD prior to index IS (HR, 2.40; 95% CI (1.79-3.20)). Compared to the patients with no-HPLD, the presence of HPLD prior to index IS increased the recurrent IS rate among DM by 88% (HR, 1.88; 95% CI (1.44-2.45)) (Figure 2).

Among patients with non-DM, the recurrent IS rate among patients with HPLD and HTN was more than two times higher than that in those with no-HPLD or HTN prior to index IS (HR, 2.80; 95% CI (2.00-3.90)), (HR, 2.20; 95% CI (1.53-3.14)) respectively, while receiving antihyperlipidemic medication among IS patients with non-DM decreased recurrent IS by 43.3% (HR, 0.433; 95% CI (0.65-0.28)) (Figure 2).

Predictive Performance Evaluation of the Final Developed TTE Model Among Patients With DM and Patients With Non-DM (TTE Model 1&2 Internal Validation)

**Figure 3:**
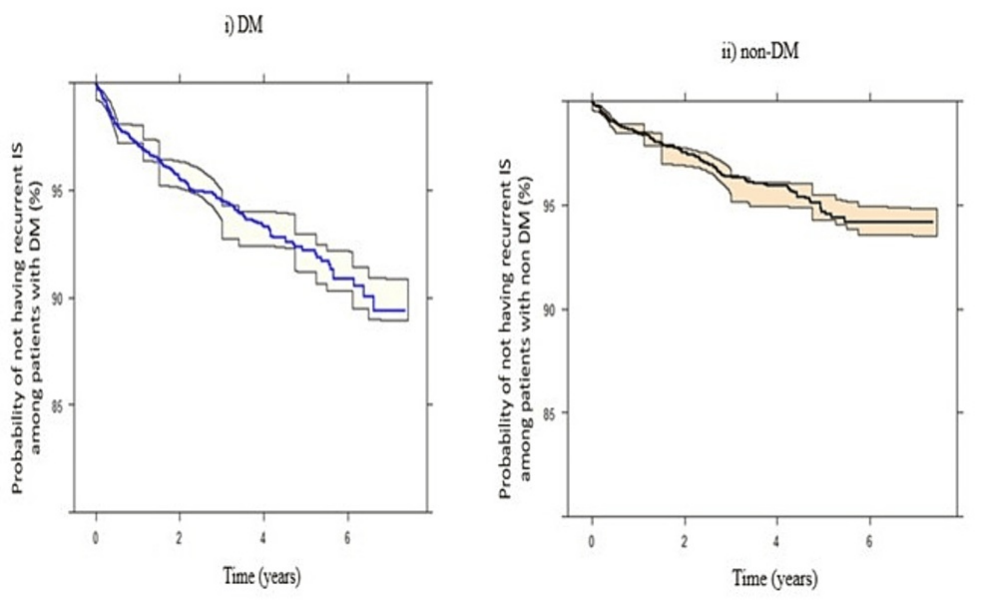
Kaplan-Meier VPCs for i) patients with diabetes (probability of not having recurrent ischemic stroke among patients with diabetes), ii) patients with non-diabetes (probability of not having recurrent ischemic stroke among patients with non-diabetes), throughout different time intervals

Kaplan-Meier VPCs for recurrent IS among DM and non-DM patients after index IS showed good predictions and indicated that the final model described the observed data adequately for internal validation (Figure 3). The survival curve (probability of not having recurrent IS after index IS) among patients with DM decreased dramatically compared to patients with non-DM.

Temporal Validation of the Final Developed TTE Model 2 Among Patients With DM

**Figure 4:**
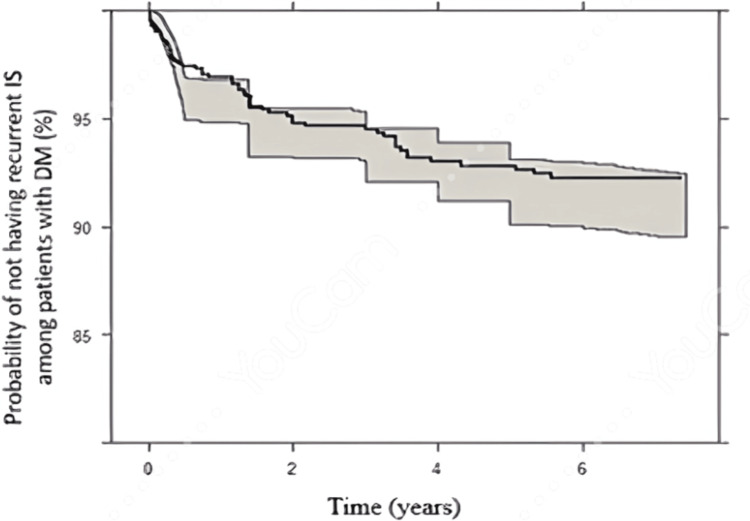
. Kaplan-Meier plots for temporal validation of the final developed model among diabetic patients throughout different time intervals

Kaplan-Meier VPCs for recurrent IS among DM patients after index IS showed good predictions, which meant that the final developed model successfully described the observed data for temporal validation (Figure 4).

Clinical Application of the Developed TTE Model Among Patients With DM (TTE 2 Model)

A calculator tool (MyReCuRIS_DM) has been created for the TTE model among patients with DM and can be opened via this link.

https://www.calconic.com/calculator-widgets/recurrent-ischemic-stroke-cal-among-patients-with-dm/63504830363df1002a572f39?layouts=true

The probability of having recurrent IS after a certain period was calculated using the calculator based on the two clinical scenarios.

Scenario 1: The probability of having recurrent IS in a DM patient with no-HPLD or IHD was estimated at 8.396% and 4.917% after one and four years of the first IS attack, respectively.

Scenario 2: The probability of having an IS recurrence for a DM patient with an IHD history was calculated as 11.403% and 18.989% after one and four years of the first IS attack, respectively.

## Discussion

It has been stated that the risk of stroke recurrence is higher (between 6-14%) within the first year after the first stroke, as compared to the recurrence risk in subsequent years (4% annually) [24]. Another study reported that the stroke recurrence incidence was the highest during the first year after the initial stroke; it was 12.8%, then declined to 6.3% during the second year and 5.1% three years after the initial stroke [25].

All studies mentioned above reported the risk of recurrent IS among the population using conventional analysis (for example, Cox regression) that could not isolate the influencing effects of risk factors. In this study, the developed TTE models incorporated the predicted hazard of recurrent IS after index IS with no influence of any risk factor as one component of the recurrent IS prognostic model. In the TTE-developed models, the baseline hazard of IS recurrence was estimated to be highest during the first six months, and even after this interval, the baseline hazard was reported as not equal to zero (Table 3). This indicates that the ‘natural history' of the IS is postulated to play a role in recurrent IS. In addition to controlling the risk factor of recurrent IS, the influence of this ‘natural history’ of IS should be considered when defining optimal secondary preventive strategies. Moreover, it was noticed that the baseline hazard of recurrent IS during the first six months among patients with DM was the highest compared to those with non-DM (Figure 1). These findings were consistent with previous findings that reported DM as an independent predictor for IS recurrence [26] and confirmed the role of the disease's natural history in IS recurrence. These findings may encourage healthcare providers to keep close monitoring and intervention, particularly for the first six months, and suggest a need for more intensive patient follow-up to ensure the efficacy of secondary preventive medications provided to patients during the first six months after the index IS.

After adding risk factors, the hazard was predicted to exponentially increase during the first three years, in agreement with the previous report [27], then decrease after three years. The difference in the hazard of recurrent IS during these intervals may be explained by better implementation of secondary stroke prevention strategies. Moreover, this indicates that early and extensive secondary IS prophylaxis, specifically during the first three years after the index IS, is paramount to preventing recurrent IS. The schedules of follow-up after the index could be modified and personalized according to each patient's risk factors. Those with more influencing risk factors may require frequent follow-up visits after the stroke and more restrictive therapy goals for controlling their concomitant diseases. The same trend was seen in a previous study, which showed that the incidence of recurrent IS for those was the highest within the first two years after IS, then decreased substantially [28]. However, in the mentioned study [28], no risk factors reported a significant association with recurrent IS incidence, indicating a multi-factorial causation of recurrent IS.

It was observed that the survival of IS patients with DM (free of recurrent IS) is dramatically decreased compared with patients with non-DM (Figure 3). This could explain the coexistence of DM with other risk factors, such as HTN or HPLD, which multiplies the IS recurrence risk among these patients [29]. More recent research found that within 3.25 years after ischaemic stroke, patients with DM have a higher risk of recurrent vascular events than patients without diabetes (the adjusted hazard ratio of diabetes was 1.50 (95% CI: 1.08-2.10) [12]. Additionally, among the 14,526 ischaemic stroke patients in the China National Stroke Registry, there were higher frequencies of recurrent stroke at the three- and six-month time points among patients with DM compared to non-DM [13]. Moreover, previous studies reported variabilities in the stroke pattern between patients with DM and non-DM. Compared to hemorrhagic stroke, IS occurs in a higher proportion among patients with DM, and the lacunar subtype is the most frequent among these patients. This may be explained by the co-existence of HTN and the increased prevalence of microvascular complications among these patients [30,31]. Moreover, the prognostic pattern among DM patients also varies from the non-DM stroke population, as DM is associated with an elevated risk of longer hospital stays, subsequent strokes, an increased risk of functional disability, and mortality [11,32]. As recurrent neurological damage is known to be serious, harder to control, and cause higher mortality compared to the first stroke [14], new strategies for management and secondary prevention are crucial to reducing recurrent IS events, especially among diabetic patients [14].

HPLD was found to increase the risk of recurrent IS among patients with and without DM. This could be explained by the angiopathy resulting from atherosclerotic plaque [29]. High cholesterol promotes atherosclerosis by building up on the artery walls and thus narrowing the arteries. Thus, blocking the blood flow predisposes to IS [33]. IHD was recorded as a significant covariate among patients with DM (Figure 2). This could be explained by the fact that IHD and IS share similar pathophysiology, mainly because atherosclerosis is manifested in both conditions [34], which became more prominent among patients with DM due to DM-associated angiopathy [35]. Patients who have atherosclerosis become at risk for acute stroke. In both cases, a sudden circulation change arises, and as a result, the blood supply decreases to some parts of the brain or heart [34].

HTN was reported as an independent predictor for recurrent IS in the TTE model among patients with non-DM, in agreement with Khanevski et al. [36]. A previous study reported a significant reduction in recurrent IS risk with BP long-term control [37]. Chronic HTN alters vascular morphology and blood flow and elevates susceptibility to neurological deterioration [38]. The co-existence of HTN could explain the non-significance of HTN among DM patients in this study among IS patients with DM [29] before the stroke.

Antihyperlipidemic agents decreased the hazard of recurrent IS among patients with non-DM. However, no significant association was reported among patients with DM, in agreement with Zhang et al.'s [15] findings that statin use was associated with lower stroke recurrence in non-diabetic patients after AIS. However, no definite association between inpatient statin use and stroke recurrence among Chinese patients with DM was found [15]. These results may support the previous results that reported increased IS recurrence among patients with DM, so regular treatment with statins may be insufficient. These patients should probably be considered to receive additional care and improve secondary preventative strategies. Prior studies illustrated that statins might accelerate DM progression through molecular mechanisms that impact IR and carbohydrate cellular metabolism [39,40]. Thus, whether the efficacy of the antihyperlipidemic agent was compromised by DM influence or affected by the statin drug is unclear, and further studies are needed to verify.

Although developing a new and potentially better model might be tempting for researchers, most developed models will never be utilized due to a lack of external or temporal validation. Temporal validation of existing models may help bridge the gap between the development and implementation of the prediction model [41]. One strength of the developed TTE model among patients with DM in this study is that it is temporally validated among many subjects, translating to a good model predictability performance (Figure 4).

The developed TTE models may describe the recurrent IS baseline hazard, its distribution during different time intervals, and additionally quantify the influence of concurrent diseases on this hazard. These findings may guide clinicians to keep close monitoring and intervention, particularly during the first six months after IS, and suggest a need for more intensive patient screening and new strategies for secondary prevention among IS patients with DM. This may help in future preventive strategies according to each patient's estimated risk of IS recurrence.

Limitations

The data was collected, respectively, from the National Stroke Registry of Malaysia. Thus, important data on possible influential risk factors for stroke, such as hormonal replacement therapy, diet, and lifestyle, were not available. Additionally, the data usage was limited by high missing values, as its handling with imputation may not be feasible. Nevertheless, this model may highlight the importance of categorizing IS patients according to the quantified hazard of IS recurrence and the consequences of the coexistence of more than one risk factor, thus tailoring better preventive strategies. Moreover, this model highlighted the importance of frequent follow-up, particularly in the first year; thus, these findings may make a positive shift in the follow-up schedule among DM patients.

## Conclusions

The developed TTE models revealed that the baseline hazard of recurrent IS was the highest during the first six months after index IS. Moreover, survival, defined as being free from recurrent IS, was the lowest among IS patients with DM compared with non-DM. Additionally, receiving antihyperlipidemic medications failed to demonstrate a significant association with reducing IS recurrence among IS patients with DM, suggesting a need for new strategies for secondary prevention among these patients. These results may encourage clinicians to keep close monitoring and intervention, especially during the first six months after IS, and suggest a need for more intensive patient screening and new strategies for secondary prevention among IS patients with DM. This may help in future preventive strategies according to each patient's estimated risk of IS recurrence.

## References

[1] Roth GA, Mensah GA, Johnson CO (2020). Global burden of cardiovascular diseases and risk factors, 1990-2019: update from the GBD 2019 study. J Am Coll Cardiol.

[2] Varona JF (2010). Long-term prognosis of ischemic stroke in young adults. Stroke Res Treat.

[3] Donnan GA, Fisher M, Macleod M, Davis SM (2008). Stroke. Lancet.

[4] Davis SM, Donnan GA (2012). Secondary prevention after ischemic stroke or transient ischemic attack. N Engl J Med.

[5] Boulanger M, Béjot Y, Rothwell PM, Touzé E (2018). Long-term risk of myocardial infarction compared to recurrent stroke after transient ischemic attack and ischemic stroke: systematic review and meta-analysis. J Am Heart Assoc.

[6] Wild S, Roglic G, Green A, Sicree R, King H (2004). Global prevalence of diabetes: estimates for the year 2000 and projections for 2030. Diabetes Care.

[7] Liao CC, Shih CC, Yeh CC, Chang YC, Hu CJ, Lin JG, Chen TL (2015). Impact of diabetes on stroke risk and outcomes: two nationwide retrospective cohort studies. Medicine (Baltimore).

[8] Khan MA, Hashim MJ, King JK, Govender RD, Mustafa H, Al Kaabi J (2020). Epidemiology of type 2 diabetes - global burden of disease and forecasted trends. J Epidemiol Glob Health.

[9] Aziz S, Sheikh Ghadzi SM, Abidin NE (2019). Gender differences and risk factors of recurrent stroke in type 2 diabetic Malaysian population with history of stroke: the observation from Malaysian national neurology Registry. J Diabetes Res.

[10] Albitar O, Harun SN, Abidin NE (2020). Predictors of recurrent ischemic stroke in obese patients with type 2 diabetes mellitus: a population-based study. J Stroke Cerebrovasc Dis.

[11] Shou J, Zhou L, Zhu S, Zhang X (2015). Diabetes is an independent risk factor for stroke recurrence in stroke patients: a meta-analysis. J Stroke Cerebrovasc Dis.

[12] Venketasubramanian N (2021). Recurrent vascular events in ischaemic stroke patients with diabetes. Ann Acad Med Singap.

[13] Jia Q, Zhao X, Wang C (2011). Diabetes and poor outcomes within 6 months after acute ischemic stroke. The China National Stroke Registry. Stroke.

[14] Zhuo Y, Wu J, Qu Y (2020). Clinical risk factors associated with recurrence of ischemic stroke within two years: a cohort study. Medicine (Baltimore).

[15] Zhang X, Li SY, Jing J (2019). Inpatient statin use and stroke recurrence in patients with or without diabetes mellitus. Neurol Res.

[16] Foulkes MA, Sacco RL, Mohr JP, Hier DB, Price TR, Wolf PA (1994). Parametric modeling of stroke recurrence. Neuroepidemiology.

[17] Elhefnawy ME, Sheikh Ghadzi SM, Tangiisuran B (2021). Population-based study comparing predictors of ischemic stroke recurrence after index ischemic stroke in non-elderly adults with or without diabetes. Int J Gen Med.

[18] Truelsen T, Heuschmann PU, Bonita R (2007). Standard method for developing stroke registers in low-income and middle-income countries: experiences from a feasibility study of a stepwise approach to stroke surveillance (STEPS Stroke). The Lanc Neuro.

[19] Aziz ZA, Lee YY, Sidek NN, Ngah BA, Looi I, Hanip MR, Basri HB (2016). Gender disparities and thrombolysis use among patient with first-ever ischemic stroke in Malaysia. Neurol Res.

[20] Austin PC, van Klaveren D, Vergouwe Y, Nieboer D, Lee DS, Steyerberg EW (2016). Geographic and temporal validity of prediction models: different approaches were useful to examine model performance. J Clin Epidemiol.

[21] Holford N (2013). A time to event tutorial for pharmacometricians. CPT Pharmacometrics Syst Pharmacol.

[22] Katsube T, Khandelwal A, Hooker AC, Jonsson EN, Karlsson MO (2012). Characterization of stepwise covariate model building combined with cross-validation. Uppsala Universitet.

[23] Elhefnawy ME, Ghadzi SM, Albitar O (2022). Predictor naïve temporal baseline hazard of recurrent ischemic stroke. Research Square.

[24] de la Cámara AG, Arche JF, Vivas PF (2013). Recurrence after a first-ever ischemic stroke development of a clinical prediction rule. Res Neurol Int J.

[25] Buenaflor FGB, Navarro JC, Lara KJA, Venketasubramanian N (2017). Recurrence rate of ischemic stroke: a single center experience. Austin J Cerebrovasc Dis & Stroke.

[26] Zhang L, Li X, Wolfe CD, O'Connell MD, Wang Y (2021). Diabetes as an independent risk factor for stroke recurrence in ischemic stroke patients: an updated meta-analysis. Neuroepidemiology.

[27] Stahmeyer JT, Stubenrauch S, Geyer S, Weissenborn K, Eberhard S (2019). The frequency and timing of recurrent stroke: an analysis of routine health insurance data. Dtsch Arztebl Int.

[28] Buenaflor FG (2017). Recurrence rate of ischemic stroke: a single center experience. Journal of the Neurological Sciences.

[29] Chen W, Pan Y, Jing J (2017). Recurrent stroke in minor ischemic stroke or transient ischemic attack with metabolic syndrome and/or diabetes mellitus. J Am Heart Assoc.

[30] Vaidya V, Gangan N, Sheehan J (2015). Impact of cardiovascular complications among patients with type 2 diabetes mellitus: a systematic review. Expert Rev Pharmacoecon Outcomes Res.

[31] Tun NN, Arunagirinathan G, Munshi SK, Pappachan JM (2017). Diabetes mellitus and stroke: a clinical update. World J Diabetes.

[32] Capes SE, Hunt D, Malmberg K, Pathak P, Gerstein HC (2001). Stress hyperglycemia and prognosis of stroke in nondiabetic and diabetic patients: a systematic overview. Stroke.

[33] Menet R, Bernard M, ElAli A (2018). Hyperlipidemia in stroke pathobiology and therapy: insights and perspectives. Front Physiol.

[34] Soler EP, Ruiz VC (2010). Epidemiology and risk factors of cerebral ischemia and ischemic heart diseases: similarities and differences. Curr Cardiol Rev.

[35] Mateshuk-Vatseba L, Savka I, Tsytovskyi M (2022). Angiopathy as a cause of structural organ changes under experimental conditions in diabetes mellitus. Journal of International Dental and Medical Research.

[36] Khanevski AN, Bjerkreim AT, Novotny V, Naess H, Thomassen L, Logallo N, Kvistad CE (2019). Recurrent ischemic stroke: incidence, predictors, and impact on mortality. Acta Neurol Scand.

[37] McGurgan IJ, Kelly PJ, Turan TN, Rothwell PM (2022). Long-term secondary prevention: management of blood pressure after a transient ischemic attack or stroke. Stroke.

[38] Li Y, Shen Q, Huang S, Li W, Muir ER, Long JA, Duong TQ (2015). Cerebral angiography, blood flow and vascular reactivity in progressive hypertension. Neuroimage.

[39] Maki KC, Ridker PM, Brown WV, Grundy SM, Sattar N, The Diabetes Subpanel of the National Lipid Association Expert Panel (2014). An assessment by the Statin Diabetes Safety Task Force: 2014 update. J Clin Lipidol.

[40] Axsom K, Berger JS, Schwartzbard AZ (2013). Statins and diabetes: the good, the bad, and the unknown. Curr Atheroscler Rep.

[41] Ramspek CL, Jager KJ, Dekker FW, Zoccali C, van Diepen M (2021). External validation of prognostic models: what, why, how, when and where?. Clin Kidney J.

